# HLA-G 14 bp polymorphism: Implications and its associated risk to malaria severity among iron-fortified Ghanaian infants and young children

**DOI:** 10.1371/journal.pone.0322524

**Published:** 2025-09-15

**Authors:** Adjoa Tetteh Danquah, David Courtin, Samuel Asamoah Sakyi, Gideon Kwesi Nakotey, Samuel Kofi Tchum, Nana Afriyie Duah, Alisé Lagrave, Christian Sewor, Benjamin Amoani

**Affiliations:** 1 Department of Microbiology and Immunology, School of Medical Sciences, University of Cape Coast, Cape Coast, Ghana; 2 UMR 261 MERIT, Université de Paris, Institut de Recherche pour le Développement (IRD), Paris, France; 3 Department of Molecular Medicine, School of Medical Sciences, Kwame Nkrumah University of Science and Technology, Kumasi, Ghana; 4 Department of Medical Laboratory Technology, Central College of Science and Technology, Agona Swedru, Ghana; 5 Kintampo Health Research Centre, Ghana Health Service, Kintampo, Ghana; 6 Department of Biomedical Science, School of Allied Health Sciences, University of Cape Coast, Cape Coast, Ghana; Fundação Oswaldo Cruz Centro de Pesquisas René Rachou: Fundacao Oswaldo Cruz Instituto Rene Rachou, BRAZIL

## Abstract

**Background of the study:**

Malaria is a paramount health concern mostly among infants and young children. The World Health Organization recommends iron fortification for children with iron deficiency anaemia living in malaria-endemic regions like Ghana. However, the intricate interplay between genetic polymorphisms and nutritional interventions in malaria susceptibility and severity remains unclear. The Human Leukocyte Antigen-G (*HLA-G)* locus within the Major Histocompatibility Complex (MHC) genes has surfaced as a critical player in regulating immune responses and influencing disease outcomes. Therefore, we aimed to evaluate the effect of *HLA-G 14* bp polymorphism and its associated risk of malaria severity among Ghanaian infants and young children on iron fortification.

**Methodology:**

This secondary double-blinded cluster randomized controlled trial involved 432 archival samples from the Tain District and Wenchi Municipal in the Bono Region. Participants aged between 6 and 36 months and consuming semi-solid foods were recruited in the study while children with malaria infection or other known medical conditions were excluded. Capillary blood samples were taken for anaemia determination using a haematology autoanalyzer, malaria infection status, and parasitaemia were assessed via microscopy, and *HLA-G 14* bp polymorphism using PCR. Hardy-Weinberg equilibrium and multivariate regression models were used to analyze the data obtained.

**Results:**

The research findings indicate that among the iron-fortified children with *HLA-G, 14* bp + /- and 14 bp-/- variants are likely to develop severe malaria. Also, the *HLA-G *14** bp + /- variant was linked to a higher risk of anaemia development among participants who received iron supplements.

**Conclusion:**

The study results indicated that iron-fortified individuals carrying the *HLA-G 14* bp insertion/deletion polymorphism have an elevated risk of developing severe malaria, which in turn predisposes them to anaemia.

## Introduction

Africa bears the heaviest burden of malaria globally, accounting for 96% of all malaria-related deaths and 95% of cases [1]. Among the affected population, the highest at-risk group are under five children with 80% of fatalities occurring in this age group [1]. The greatest significant morbidity and death is known to be caused by *Plasmodium falciparum* within the Sub-Saharan region [2]. The latest World Malaria Report (WMR) reveals that the number of malaria cases rose from 244 million in 2021–249 million in 2022 [1]. The African Regions continue to bear an unevenly high burden of malaria [1]. According to the WMR, in 2022, around 94% of all malaria infections and 95% of deaths occurred in this region [1]. Alarmingly, about 78% of these deaths were children under five years old^1^. The Severe Malaria Observatory listed Ghana as one of the 15 countries with a high malaria burden, contributing 2.1% of cases and 1.9% of fatalities worldwide [3].

The intricate interplay between genetic factors and infectious diseases has garnered significant interest, with the major histocompatibility complex (MHC) assuming a central role [4]. Within the MHC genes, the human leukocyte antigen G (*HLA-G*) locus has surfaced as a critical player in regulating immune responses and influencing disease outcomes [4]. The *HLA-G* is a non-classical human leukocyte antigen (HLA), which produces tolerogenic and immunosuppressive properties [5].

Several studies have reported that, regarding malaria, the role of *HLA-G* has drawn significant interest due to its immunomodulatory properties [6,7]. The 14 bp deletion of *HLA-G* in malaria correlates with elevated circulating amounts of soluble HLA-G, which has significant immunosuppressive capabilities via interacting with inhibitory receptors on immune cells. This may modify the balance between pro-inflammatory and anti-inflammatory responses, influencing the synthesis of cytokines such as IL-10, TGF-β, and IFN-γ. Moreover, elevated sHLA-G levels may hinder efficient parasite elimination. Specifically, the 14 bp insertion/deletion variation in the 3’ untranslated region (3’UTR) of the *HLA-G* gene has been the subject of intensive research [8]. This genetic variation affected *HLA-G* alternative splicing and *HLA-G* messenger RNA (mRNA) stability. The homozygous genotype 14 bp insertion (+/+) was associated with lower mRNA and soluble HLA-G levels [9] than the genotypes 14 bp insertion (+/-) or (-/-) [10] and therefore could play a crucial role in determining susceptibility to malaria infection, influencing disease severity, and contributing to the development of anaemia.

Over the past decades, global efforts to address malnutrition have intensified, with food fortification programs gaining prominence, especially in the malaria endemic under international guidelines [11]. These initiatives primarily target micronutrient deficiencies, including iron, an essential element for haemoglobin synthesis and red blood cell formation [12]. However, emerging evidence indicates that iron supplementation could potentially worsen malaria infections among iron-replete infants and young children living in malaria-endemic areas. The mechanism behind this lies in the increased availability of free heme, a potent pro-oxidant and a substrate that supports *Plasmodium* growth [13]. On the one hand, studies have demonstrated that iron-depleted children receiving iron supplements have enhanced protection against malaria and anaemia [14]. Conversely, other studies have indicated that administering iron supplements to children increases their likelihood of contracting malaria [15]. It is still unknown if variations in the *HLA-G 14* bp gene among Ghanaian children receiving iron supplementation could influence malaria disease outcome and severity. Thus, this study evaluated the effect of *HLA-G 14* bp polymorphism and its associated risk of malaria severity between iron and non-iron-fortified Ghanaian children.

## Methodology

### Study design and site

This study was a double-blinded cluster randomized control trial conducted in the Tain District and Wenchi municipal in the Bono Region of Ghana from 3/04/2010–6/07/2010. Details of the study area have been reported in a previous study^16^. These study areas are situated between latitudes 7° 30′ South and 7° 15′ North and longitudes 2° 17’ West and 1° 55′ East [16] where diverse forms of farming are the major sources of income within these two communities. A structured questionnaire was used to obtain socio-demographic characteristics from study participants.

### Ethical considerations and recruitment of study participants

**T**he ethics committees of the Ghana Health Service, Food and Drugs Authority of Ghana, Kintampo Health Research Centre and Hospital for Sick Children (SickKids), Canada reviewed and approved the study protocol. This current study is a secondary analysis of data from the trial registered at ClinicalTrials.gov (Identifier: NCT01001871). The trial was overseen by an independent Data and Safety Monitoring Board (DSMB), constituted in October 2009 and held three meetings during the trial. Members of the DSMB included international and local health policymakers with expertise in randomized controlled trials, nutrition, paediatrics, statistics and social sciences. The DSMB’s statistician summarized the compiled outcome data at the end of the recruitment phase and halfway through the intervention stage for any serious adverse effects. The children’s primary caregivers consented to participate in the study through a written consent form. For the interim analysis, if there were any serious adverse events (i.e. hospital admissions or deaths) in the iron group than the non-iron group, the agreement a *priori* was that the study would be terminated.

Using Cochran’s formula for sample size determination, a total of 432 participants aged between 6–36 months comprising 244 iron-supplemented and 188 non-iron-supplemented groups whose parents consented to the study were enrolled. The iron group received micronutrient powder (MNP) consisting of iron (12.5 mg), ascorbic acid (30 mg), zinc (5 mg), and vitamin A (400 mg) while the non-iron group received the MNP supplement without iron. The participants were followed for six months, and capillary blood samples were taken between March and April 2010. Participants’ visits to a healthcare facility were recorded using study identification cards (issued to caregivers at enrolment). Blood samples were collected from recruited children who presented with the symptoms of fever (axillary temperature >37.5°C or recent fever within 48 hours) or those admitted to a healthcare facility. The study excluded children with severe anaemia (haemoglobin (Hb) level <7.0 g/dL), acute malnourishment (weight-for-length score<−3.0), those who had taken iron supplements within the past 6 months, and children with chronic illness (e.g., HIV, congenital abnormalities, etc.). Insecticide bed nets (IBN) were provided for participants during this period described elsewhere [15]. Children with confirmed malaria were given first-line antimalarial treatment consisting of artesunate and amodiaquine or artemether and lumefantrine as described in detail elsewhere [15].

### Study procedure

Approximately 200–500 μL of heel or finger blood samples were collected from the participants into sterile ethylene diamine tetra-acetic acid (EDTA) microtubes. Briefly, 5 μL of blood sample was used for malaria screening using malaria PF RDT (Paracheck® Orchid Biomedical Systems, Goa, India) cassette. For malaria microscopy, thick and thin film blood smears were used to confirm positive test findings and parasite density. Blood samples that tested positive were classified as either uncomplicated or severe malaria, following the WHO guidelines as described in details elsewhere [1]. As per the guidelines, uncomplicated malaria is the evidence of clinical features of malaria with confirmation by laboratory testing and no cerebral involvement or other organ dysfunction; severe malaria was categorized as the presence of clinical features of malaria and confirmed by laboratory testing with cerebral involvement and other organ dysfunction (severe anaemia, hypoglycaemia, renal failure, severe dehydration, and respiratory acidosis) [17]. Anaemia levels were determined through complete blood cell count using a haematology autoanalyzer (Horiba ABX Micros 60-ot-ct-os-cs) as described elsewhere [14].

The double salt precipitation method was used to extract genomic DNA from the blood samples with slight modifications per the manufacturer’s manual for polymerase chain reaction (PCR) analysis [18]. PCR was used as the gold standard technique for HLA-G 14 bp insertion/deletion genotyping because of its great specificity, sensitivity, and dependability in differentiating between the various genotypes. Amplification of *HLA-G* 14 bp insertion/deletion variant (with protein accession ID P17693 and polymorphism ID rs 66554220) was performed using the forward primer 5′GTGATGGGCTGTTTAAAGTGTCACC-3′ and reverse primer 5′-GGAAGGAATGCAGTTCA GCATGA-3′. The PCR amplifications were done following this protocol: 10x Taq buffer, 2.5 mM 2’- deoxyribonucleotide 5’-triphosphate (dNTP) mix, 3 μL of DNA, 7.13 μL of nuclease-free water, 100 μM of each primer, 25 mM of MgCl_2_ and 1.25 U/ μL Taq polymerase (Promega, Madison, CA, USA) in a final volume of 15 μL. The PCR amplification was done under the following conditions: 95°C for initial denaturation for 5 min, followed by 33 cycles at 94 °C for 60 s, 61 °C for 60 s, 72 °C for 1 min and a final extension at 72 °C for 10 min.

5 µL of the PCR product was then migrated in 3% agarose gel and stained with 0.5 µg/ mL ethidium bromide (Life Technologies Co, USA) for 40 min at 100 volts. The agarose gel electrophoresis was used to confirm the success of the amplification. A standard DNA of known molecular weight (100 base pairs) was loaded to determine each DNA fragment’s molecular weight, which was then amplified by electrophoresis. The UV-illumination gel document was used to view the results of the electrophoresis. Direct counting was used to determine the total number of 14 bp insertions or deletions and both insertion and deletion alleles. Band sizes of 210 bp, 225 bp, both 210 bp and 225 bp indicate Deletion, Insertion and both Insertion/Deletion, respectively.

### Statistical analysis

The data obtained were imported into SPSS (IBM, Version 26., Chicago, USA) and Stata version 17 (SE). The Hardy-Weinberg equilibrium (HWE) was used to estimate test allelic frequency distribution, where p and q were the only alleles in this situation, and therefore p + q = 1. The descriptive patterns in the study population were summarized as frequencies with the respective percentages and mean with their respective standard deviations. Bivariate associations were assessed via t-test for continuous variables and chi-square test for categorical variables. Logistic regression (both multinomial and binary) models were used to assess the association between gene variants, the severity of malaria, and anaemia with the respective risk ratio (RRR) and odds ratio (OR) with their corresponding 95% Confidence intervals (Cis) reported. All models were adjusted for age and sex. A p-value less than 0.05 was deemed to indicate statistical significance.

## Results

### Demographic and clinical characteristics of the study participants

This study found that for both iron and non-iron groups, the majority of the participants were males. Participants within the iron group recorded a slightly higher mean age (19.01 ± 8.43 mo) than those in the non-iron group (18.83 ± 7.97 mo). At the endline, there was a difference between the iron and the non-iron group in terms of anaemia status (p = 0.025) contrary to what was observed at baseline (p = 0.683) (Table 1**).**

**Table 1:** Demographic and Clinical Characteristics of the Study Participants.

Characteristics	Iron-Group (N = 244)	Non-Iron Group (N = 188)	p-value
**Gender**			
Male	138 (56.6)	98 (52.1)	0.359^a^
Female	106 (43.4)	90 (47.9)	
**Age (in months)**	19.01 ± 8.43	18.83 ± 7.97	0.823^b^
**Infection Status**			
Negative	107 (60.5)	70 (39.6)	0.365^a^
Uncomplicated	85 (54.5)	71 (45.5)	
Complicated	52 (52.5)	47 (47.5)	
**Anaemia Status at Baseline**			
Yes	89 (57.8)	65 (42.2)	0.683^a^
No	155 (55.8)	123 (44.2)	
**Anaemia Status at Endline**			
Yes	135 (52.1)	124 (47.9)	**0.025** ^a^
No	109 (63.0)	64 (37.0)	
**Polymorphism**			
Insertion (+/+)	99 (62.7)	59 (37.4)	0.105^a^
Insertion/Deletion (+/-)	102 (54.6)	85 (45.5)	
Deletion (-/-)	43(49.4)	44(50.6)	
**Parasitaemia**	75693 ± 130358.3	79395 ± 104243.2	0.750^b^

^a^(p-value generated using Chi-square test), ^b^ (p-value generated using T-test).

### The frequency of the 14 bp polymorphism alleles to malaria status within the Iron group

In the iron group, the *HLA-G 14* bp insertion (+/+) allele recorded a 61% frequency, while the *HLA-G 14* bp deletion (-/-) allele had a frequency of 38%. A significant variation was observed in the distribution of the various 14 bp variants when compared against malaria status (p = 0.041) (Table 2).

**Table 2:** The frequency of the 14 bp polymorphism alleles to malaria status within the Iron Group.

Type of SNP	Negative (N = 107)	Uncomplicated (N = 85)	Complicated (N = 52)	p-value
-/- (N = 43)	17 (39.5)	20 (46.5)	6 (14.0)	**0.041**
+/- (N = 102)	37 (36.3)	37 (36.3)	28 (27.5)	
+/+ (N = 99)	52 (53.5)	28 (28.3)	19 (18.2)	
**Alleles**				
+	0.67 (14.3)	0.55 (9.3)	0.63 (6.6)	
–	0.33 (7.1)	0.45 (7.7)	0.38 (4.0)	

+/+ (insertion), + /- (insertion/deletion), -/- (deletion), n (%) p-value generated using Chi-square test.

### The frequency of the 14 bp polymorphism alleles to malaria status within the Non-Iron Group

The frequency of the *HLA-G 14* bp + / + allele was 55% and that of the *HLA-G 14* bp -/- allele was 45% in the non-iron group. Despite these variations, there was no statistically significant difference (p = 0.939) in the distribution of the different 14 bp variants within the non-iron group (Table 3**).**

**Table 3:** The frequency of the 14 bp polymorphism alleles to malaria status within the Non-Iron Group.

Type of SNP	Negative (N = 70)	Uncomplicated (N = 71)	Complicated (N = 47)	p-value
-/- (N = 44)	18 (40.9)	17 (38.6)	9 (20.5)	0.939
+/- (N = 85)	31 (36.5)	31 (36.5)	23 (27.1)	
+/+ (N = 59)	21 (35.6)	23 (39.0)	15 (25.4)	
**Alleles**				
+	0.56 (7.3)	0.54 (7.7)	0.56 (5.3)	
–	0.44 (5.7)	0.46 (6.5)	0.44 (1.4)	

+/+ (insertion), +/- (insertion/deletion), -/- (deletion), n (%), p-value generated using Chi-square test).

### Association between 14 bp polymorphisms and malaria disease severity among iron fortified children

In the multinomial logistic regression model, we observed that iron-fortified children with 14 bp -/- polymorphism were at an increased risk of experiencing uncomplicated malaria (RRR = 2.56; 95% CI: 1.13, 5.77; p = 0.024) than those with 14 bp + / + . Similarly, children who carry 14 bp + /- polymorphism were at a higher risk of experiencing both uncomplicated (RRR = 2.09; 95% CI: 1.08, 4.05; p = 0.029) and complicated malaria (RRR = 2.51; 95% CI: 1.19, 5.03; p = 0.016) than those with 14 bp + / + . In the binary logistic regression model, we found that individuals with 14 bp + /- were at higher odds (RRR = 1.17; 95% CI = 0.53, 2.57; p > 0.05) of experiencing complicated malaria compared with those with 14 bp insertion although not statistically significant (Table 4).

**Table 4:** Association between 14 bp Polymorphisms and Malaria Severity among Iron Fortified Children.

Polymorphisms	Crude Analysis, RRR/OR (95% CI)	p-value	Adjusted Analysis, RRR/OR (95% CI)	p-value
	NC vs UM^a^		NC vs UM^a^	
Insertion (+/+)	Ref		Ref	
Deletion (-/-)	2.23 (1.01, 4.92)	**0.048**	2.56 (1.13, 5.77)	**0.024**
Insertion/deletion (+/-)	1.89 (0.99, 3.61)	0.053	2.09 (1.08, 4.05)	**0.029**
	**NC vs CM** ^**a**^		**NC vs CM** ^**a**^	
Insertion (+/+)	Ref		Ref	
Deletion (-/-)	1.04 (0.36, 3.04)	0.944	1.18 (0.40, 3.52)	0.767
Insertion/deletion (+/-)	2.23 (1.08, 4.60)	**0.031**	2.51 (1.19, 5.30)	**0.016**
	**UM vs CM** ^**b**^		**UM vs CM** ^**b**^	
Insertion (+/+)	Ref		Ref	
Deletion (-/-)	0.47 (0.16, 1.38)	0.170	0.41 (0.13, 1.24)	0.114
Insertion/deletion (+/-)	1.18 (0.55, 2.54)	0.678	1.17 (0.53, 2.57)	0.697

^a^Multinomial logistic regression on the effect of polymorphism type on infection status (N = 244), the model was adjusted for age and sex with Negative controls as the reference group. ^b^Binary logistic regression on the effect of polymorphism type on infection status (N = 137), the model was adjusted for age and sex with the uncomplicated group as the reference category. OR (odds ratio), RRR (Relative Risk Ratio), NC (negative control), UM (uncomplicated malaria, CM (complicated malaria).

### Association between 14 bp polymorphisms and malaria severity in non-iron fortified children

Results from the multinomial logistic regression analysis showed that compared to children in the negative control group, children who carry 14 bp + /- showed marginally relatively higher risk (RRR = 1.01; 95% CI = 0.53, 2.57; p = 0.975) of experiencing complicated malaria than those with 14 bp + / + , albeit the interval estimate contained the null value (Table 5).

**Table 5:** Association of 14 bp Polymorphisms and Malaria Disease Severity in Non-Iron Fortified Children.

Polymorphisms	Crude Analysis RRR/OR (95% CI)	p-value	Adjusted Analysis RRR/OR (95% CI)	p-value
	NC vs UM^a^		NC vs UM^a^	
Insertion (+/+)	Ref		Ref	
Deletion (-/-)	0.86 (0.35, 2.10)	0.744	0.84 (0.34, 2.08)	0.711
Insertion/deletion (+/-)	0.91 (0.42, 1.98)	0.818	0.89 (0.40, 1.94)	0.760
	**NC vs CM** ^**a**^		**NC vs CM** ^**a**^	
Insertion (+/+)	Ref		Ref	
Deletion (-/-)	0.70 (0.25, 1.98)	0.501	0.67 (0.23, 1.91)	0.451
Insertion/deletion (+/-)	1.04 (0.44, 2.44)	0.931	1.01 (0.43, 2.39)	0.975
	**UM vs CM** ^**b**^		**UM vs CM** ^**b**^	
Insertion (+/+)	Ref		Ref	
Deletion (-/-)	0.81 (0.29, 2.29)	0.694	0.87 (0.30, 2.49)	0.792
Insertion/deletion (+/-)	1.14 (0.49, 2.65)	0.765	1.22 (0.52, 2.91)	0.697

^a^Multinomial logistic regression on the effect of polymorphism type on infection status (N = 188), the model was adjusted for age and sex with Negative controls as the reference group. ^b^Binary logistic regression on the effect of polymorphism type on infection status (N = 118), the model was adjusted for age and sex with the uncomplicated group as the reference category. OR (odds ratio), RRR (Relative Risk Ratio), NC (negative control), UM (uncomplicated malaria, CM (complicated malaria).

### Association between 14 bp polymorphisms and malaria severity among all children

According to the multinomial logistic regression model we observed that compared to children in the negative control, children with 14 bp + /- were at a higher risk of experiencing complicated malaria compared with individuals with 14 bp + /+ (RRR = 1.76; 95% CI = 1.01, 3.05; p = 0.045) (Table 6).

**Table 6:** Association between 14 bp Polymorphisms and Malaria Severity among all the Children.

Polymorphisms	Crude Analysis, RRR/OR (95% CI)	p-value	Adjusted Analysis, RRR/OR (95% CI)	p-value
	NC vs UM^a^		NC vs UM^a^	
Insertion (+/+)	Ref		Ref	
Deletion (-/-)	1.53 (0.86, 2.75)	0.151	1.52 (0.85, 2.74)	0.159
Insertion/deletion (+/-)	1.45 (0.89, 2.37)	0.137	1.44 (0.88, 2.36)	0.144
	**NC vs CM** ^**a**^		**NC vs CM** ^**a**^	
Insertion (+/+)	Ref		Ref	
Deletion (-/-)	0.96 (0.46, 2.00)	0.915	1.00 (0.48, 2.08)	0.994
Insertion/deletion (+/-)	1.68 (0.97, 2.91)	0.063	**1.76 (1.01, 3.05)**	**0.045**
	**UM vs CM** ^**b**^		**UM vs CM** ^**b**^	
Insertion (+/+)	Ref		Ref	
Deletion (-/-)	0.63 (0.30, 1.32)	0.217	0.64 (0.30, 1.35)	0.237
Insertion/deletion (+/-)	1.16 (0.65, 2.05)	0.611	1.23 (0.69, 2.20)	0.477

^a^Multinomial logistic regression on the effect of polymorphism type on infection status (N = 432), the model was adjusted for age and sex with Negative controls as the reference group. ^b^Binary logistic regression on the effect of polymorphism type on infection status (N = 432), the model was adjusted for age and sex with the uncomplicated group as the reference category. OR (odds ratio), RRR (Relative Risk Ratio), NC (negative control), UM (uncomplicated malaria, CM (complicated malaria).

### Association of 14 bp polymorphism on anaemia among iron group at baseline and endline

The results showed that amongst the iron-fortified participants, children with 14 bp + /- polymorphism were at an increased higher odd of being anaemic compared to those with 14 bp -/- polymorphism (OR= 2.14; 95% CI: 1.19, 3.84; p = 0.011) (Table 7).

**Table 7:** Association of 14 bp Polymorphism on Anaemia among Iron Group at Baseline and Endline.

	Baseline				Endline			
14 bp SNP	Crude Analysis, OR (95% CI)	p-value	Adjusted Analysis, OR (95% CI)	p-value	Crude Analysis, OR (95% CI)	p-value	Adjusted Analysis, OR (95% CI)	p-value
Insertion (+/+)	Ref		Ref		Ref		Ref	
Deletion (-/-)	1.50 (0.71, 3.17)	0.284	1.65 (0.74, 3.66)	0.221	0.81 (0.39, 1.66)	0.561	0.90 (0.43, 1.89)	0.789
Insertion/deletion(+/-)	1.61 (0.90, 2.88)	0.109	1.68 (0.90, 3.14)	0.100	1.95 (1.11, 3.45)	**0.021**	2.14 (1.19, 3.84)	**0.011**

Binary logistic regression of the effect of polymorphism type on anaemia status at baseline (N = 244), the model was adjusted for age and sex, OR (Odds Ratio).

### Association of 14 bp polymorphism on anaemia among non-iron group at baseline and endline

Results for the non-iron group showed are largely null association with respect to the impact of 14 bp polymorphisms on baseline and endline anaemic status (Table 8).

**Table 8:** Association of 14 bp Polymorphism on Anaemia among Non-Iron Group at Baseline and Endline.

	Baseline				Endline			
14 bp SNP	Crude Analysis, OR (95% CI)	p-value	Adjusted Analysis, OR (95% CI)	p-value	Crude Analysis, OR (95% CI)	p-value	Adjusted Analysis, OR (95% CI)	p-value
Insertion (+/+)	Ref		Ref		Ref		Ref	
Deletion(-/-)	1.58 (0.70, 3.57)	0.275	1.42 (0.61, 3.29)	0.414	0.99 (0.43, 2.26)	0.984	0.90 (0.39, 2.09)	0.810
Ins/del (+/-)	1.18 (0.59, 2.41)	0.650	1.08 (0.52, 2.25)	0.833	0.99 (0.49, 2.00)	0.978	0.93 (0.46, 1.90)	0.844

Binary logistic regression of the effect of polymorphism type on anaemia status at endline (N = 188), the model was adjusted for age and sex, OR (Odds Ratio).

## Discussion

Overall, we observed that among the iron-fortified children, HLA-G 14 bp + /- and 14 bp-/- variants were associated with a higher likelihood of developing severe malaria. Similarly, the HLA-G 14 bp + /- variant was associated with higher odds of anaemia among participants who received iron supplements. Similar studies also found that HLA-G 14 bp variants were linked to increased severe malaria risk and higher anaemia odds in iron-supplemented children, highlighting genetic influences on health outcomes [19,20]. Since there is little to no evidence of the association between *HLA-G 14* bp polymorphism and malaria infection severity, susceptibility, and anaemia within the Ghanaian population, our study provides much needed evidence regarding this association.

In this study, we observed the prevalence of anaemia increased among study participants at the endline compared to the baseline in both groups, with the cases being slightly higher in the iron group than in the non-iron group. This could be due to iron overload, resulting in the circulation of free heme which supports *Plasmodium* growth [13]. The combination of iron intake, environmental factors, genetic predisposition, and underlying health conditions may elevate the risk of anaemia and susceptibility to severe malaria [21].

Moreover, we observed significant variations in the frequencies of three specific single nucleotide polymorphisms (SNPs) among the children who received iron fortification with 14 bp insertion recording the highest frequency. This result is contrary to a study conducted in Brazil which reported a nearly equal distribution of 14 bp insertions, deletions and insertions/deletions [22]. Our findings indicate that genetic differences in individuals’ responses to interventions, such as iron fortification, may serve as predictors for their susceptibility to malaria infection.

In addition, polymorphisms among the non-iron group did not appear to be associated with malaria severity for children with 14 bp -/- and both +/- polymorphisms. *HLA-G 14* bp + /- polymorphism might not be a common genetic factor in the development of diverse immunological diseases, and separate pathogenic processes may be involved [23]. Despite this observation, it is possible that the progression of malaria status from uncomplicated to complicated could be due to genetic expressions [24]. A similar study reported that severe *P. falciparum* malaria episodes were more common in children with the *HLA-G* 3′ UTR-03 haplotype, which carries the 14 bp deletion allele. Contrary to results observed in the non-iron group, we observed that amongst iron-fortified participants, children with 14 bp + /- polymorphism had an increased risk of experiencing complicated malaria compared to those with 14 bp insertion. This observation agrees with the assertion that iron status could alter an individual’s immune response to infections [25,26]. The immunological system microenvironment may influence the outcome of infection, as shown by HLA-G expression [27]. The finding that iron-fortified infants with the HLA-G 14 bp + /- and 14 bp-/- variations showed increased vulnerability to severe malaria indicates the potential need for tailored dietary therapy. This is especially pertinent since the World Health Organization advocates for iron supplementation in children suffering from iron deficiency anemia in malaria-endemic areas [28]. Conducting HLA-G 14 bp polymorphism screening prior to commencing iron supplementation may effectively identify children at increased risk for severe malaria complications. Therefore, it is necessary to create stratified iron supplementation recommendations that take genetic susceptibility variables into account.

In the analysis relating to the entire study population, we observed that children with 14 bp + /- had a higher risk of experiencing complicated malaria than individuals with 14 bp + / + . In various populations studied, the presence of the 14 bp INS/DEL polymorphism consistently correlates with increased susceptibility to more severe forms of malaria [29,30]

Finally, we observed that iron-fortified participants with 14 bp + /- polymorphism had significantly increased odds of being anaemic (p < 0.05) compared to those with 14 bp -/- polymorphism after adjusting for age and sex. In regions where malaria is prevalent, the infection significantly contributes to anaemia by causing the breakdown of both infected and uninfected red blood cells [13]. This process is marked by transient abnormalities in bone marrow function [28]. *HLA-G*- Ig-like transcript 2 (ILT2) interaction has been reported to cause a decrease in bone marrow B cell function, leading to acquired aplastic anaemia [31]. We hypothesized that in people with iron fortification, the immunosuppressive effects of elevated sHLA-G levels (linked to 14 bp + /- and -/- genotypes) coupled with enhanced iron availability foster a conducive environment for parasite multiplication. The consequently increased parasite burden adds to severe malaria, which leads to hemolysis, dyserythropoiesis, and finally anemia.

While our study provides enough evidence regarding the association between HLA-G polymorphism and malaria severity in an interventional group, questions about the potential risks of iron supplementation in genetically susceptible populations remain unclear, necessitating further investigation to clarify these relationships and optimize health strategies for vulnerable groups. Future studies should explore the molecular pathways via which *HLA-G* variations regulate immunological responses to malaria in iron-supplemented people.

The use of a rigorous randomized controlled trial design and analysis of both genetic and nutritional intervention factors in malaria outcomes are major strengths of this study. However, it is important to note that, the relatively small sample size limiting statistical power for subgroup analyses and, the single geographical region which may limit generalizability to other malaria-endemic areas are the limitations of this study.

## Conclusion

This study revealed that people who carry the *HLA-G 14* bp insertion/deletion polymorphism are more susceptible to severe malaria, which increases their vulnerability to anaemia. The results of this study underline the importance of *HLA-G 14* bp + /- variant screening before any iron supplements in children in malaria-endemic areas.

## Supporting information

S1 Appendix Dataset for the Iron supplement group participants. (XLSX)

S2 Appendix Dataset for the Non-Iron supplement group participants. (XLSX)
